# Acute Encephalopathy and Severe Hypercalcemia as the Initial Presentation of a Large Parathyroid Hormone-Related Protein (PTHrP)-Secreting Pancreatic Neuroendocrine Tumor: A Case Report

**DOI:** 10.7759/cureus.92717

**Published:** 2025-09-19

**Authors:** Katherine M Collamore, Parul Jandir, Jennifer Hashem, Alejandro Cruz Ponce, Michael Levitt

**Affiliations:** 1 Hematology and Oncology, Hackensack Meridian School of Medicine, Nutley, USA; 2 Hematology and Oncology, Jersey Shore University Medical Center, Neptune, USA; 3 Internal Medicine, Jersey Shore University Medical Center, Neptune, USA

**Keywords:** acute metabolic encephalopathy, large abdominal mass, pancreatic neuroendocrine tumor (pnet), parathyroid hormone-related peptide (pthrp), pth-related hypercalcemia of malignancy, pthrp-secreting tumors, severe hypercalcemia

## Abstract

Pancreatic neuroendocrine tumors (pNETs) are rare neoplasms that present with diverse clinical manifestations depending on their secretory activity. Paraneoplastic hypercalcemia due to parathyroid hormone-related peptide (PTHrP) secretion by pNETs is an uncommon but serious complication. We describe the case of a 67-year-old female with a past medical history of multiple sclerosis and uveitis presenting with acute metabolic encephalopathy due to profound hypercalcemia. Laboratory workup revealed a corrected calcium of 22.4 mg/dL and PTHrP of 327 pg/mL. Imaging demonstrated a new 15 cm pancreatic mass with adrenal nodules suspicious for metastatic disease. Pathology from a percutaneous biopsy confirmed a well-differentiated pNET with a Ki-67 index of ~15%. The patient’s mental status improved following treatment of hypercalcemia with intravenous hydration, calcitonin, zoledronic acid, and urgent hemodialysis. She was started on lanreotide therapy for pNET treatment. This case highlights the importance of considering pNETs in the differential diagnosis of unexplained hypercalcemia, as timely recognition and management can reverse neurologic dysfunction and improve outcomes. Awareness of this rare presentation may facilitate earlier diagnosis and treatment of underlying neuroendocrine malignancies.

## Introduction

Neuroendocrine tumors (NETs) are rare neoplasms originating from neuroendocrine cells and demonstrate a wide spectrum of clinical and biochemical profiles depending on their anatomical site and secretory activity. Among these, pancreatic neuroendocrine tumors (pNETs) comprise 5% of all pancreatic tumors and 7% of all NETs [1]. These tumors are categorized as functional, meaning they secrete pancreatic hormones such as insulin or glucagon, or nonfunctional, meaning they do not secrete hormones [2]. Nonfunctional tumors typically present later, with symptoms resulting from tumor burden, mass effect, or metastatic disease [3].

Paraneoplastic hypercalcemia is a rare but serious complication of pNETs, most often mediated by secretion of parathyroid hormone-related peptide (PTHrP) [4]. Hypercalcemia of malignancy is a common metabolic complication seen in up to 30% of cancer patients, particularly those with squamous cell carcinoma, renal cell carcinoma, and breast cancer [2,5-7]. Although rarely seen in NETs, recent case reports of PTHrP-producing pNETs causing severe hypercalcemia requiring intensive treatments suggest that this phenomenon may be underrecognized and clinically significant [2,6-11,12].

We present the case of a well-differentiated pNET in a patient with acute encephalopathy due to profound hypercalcemia from elevated PTHrP. This case underscores the importance of considering pNETs in the differential diagnosis of hypercalcemia of unclear etiology. This report adds to growing evidence of the diverse clinical manifestations of PTHrP-secreting pNETs, emphasizing the need for clinical vigilance and timely diagnostic workup in patients with unexplained hypercalcemia and neurologic symptoms.

## Case presentation

Emergency department course

A 67-year-old female with a past medical history of multiple sclerosis and uveitis presented to the emergency department with confusion and slurred speech after being found down by family. The patient was agitated, required frequent redirection, and answered questions intermittently. The patient’s husband provided the majority of the initial history due to the patient’s confusion and inattention. He reported that she had poor appetite, low energy, and a 40 lb weight loss in the previous six months, as well as constipation. The husband also confirmed that the patient’s current behavior and confusion were an acute change from her baseline, which was fully oriented to person, place, and time with the ability to attend to and actively participate in conversation. Initial vital signs in the emergency department are shown in Table 1.

**Table 1:** Initial vital signs on arrival to the emergency department. The table details the vital signs obtained upon the patient’s arrival to the emergency department. They are significant for tachycardia (heart rate >/= 100 beats per minute) and hypertension (blood pressure >/= 130/80 mmHg), with a normal respiratory rate measured in breaths per minute and a normal oxygen saturation (SpO_2_).

Vital signs	
Heart rate	112 beats/minute
Blood pressure	142/81 mmHg
Respiratory rate	17 breaths/minute
Oxygen saturation (SpO_2_)	97%

Significant laboratory results in the emergency department are shown in Table 2.

**Table 2:** Laboratory values of significance from the emergency department. The table displays significant laboratory values from tests conducted in the emergency department upon the patient’s initial presentation. They are significant for leukocytosis (white blood cell count > 11,000/µL) with a left shift (neutrophils >70%), mild anemia (hemoglobin <12 g/dL), kidney dysfunction evidenced by elevated creatinine (>1.02 mg/dL) and accompanied by electrolyte abnormalities, including hypermagnesemia (magnesium >2.6 mg/dL) and severe hypercalcemia (calcium >>10.4 mg/dL). Additionally, results showed muscle injury evidenced by elevated creatinine kinase (>145 IU/L), liver injury evidenced by elevated liver function tests (alkaline phosphatase >116 U/L, aspartate aminotransferase >34 U/L, alanine aminotransferase >49 U/L), and chronic inflammation suggested by elevated ferritin (ferritin >270.7 ng/mL), an acute-phase reactant.

Laboratory studies	Patient results	Reference values
White blood cells	21,000/µL	4,500–11,000/µL
Neutrophil	87%	50–70%
Hemoglobin	11.8 g/dL	12–16 g/dL
Creatinine	2.22 mg/dL	0.55–1.02 mg/dL
Calcium	21.6 mg/dL	8.7–10.4 mg/dL
Corrected calcium	22.4 mg/dL	8.7–10.4 mg/dL
Magnesium	2.71 mg/dL	1.6–2.6 mg/dL
Creatinine kinase	6,016 IU/L	34–145 IU/L
Alkaline phosphatase	145 U/L	46–116 U/L
Aspartate aminotransferase	388 U/L	0–34 U/L
Alanine aminotransferase	226 U/L	10–49 U/L
Ferritin	1,502.0 ng/mL	7.3–270.7 ng/mL

**Figure 1:**
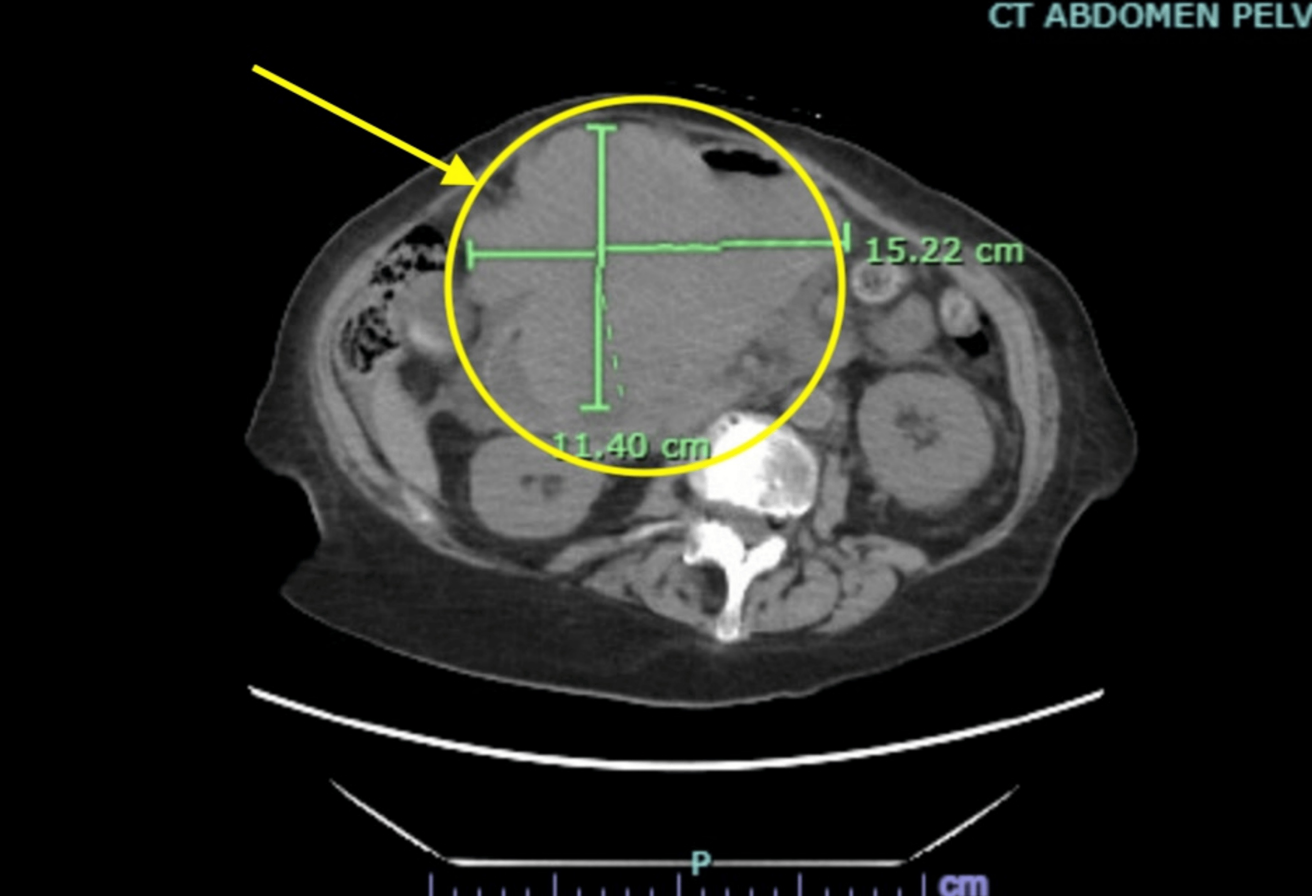
Axial view of the initial CT of the chest, abdomen, and pelvis. The image displays an axial cross-section from the patient’s CT scan of the chest, abdomen, and pelvis, obtained in the emergency department due to elevated liver function tests (see Table 2). The yellow circle and arrow indicate a large abdominal mass measuring 11.40 cm × 15.22 cm.

**Figure 2:**
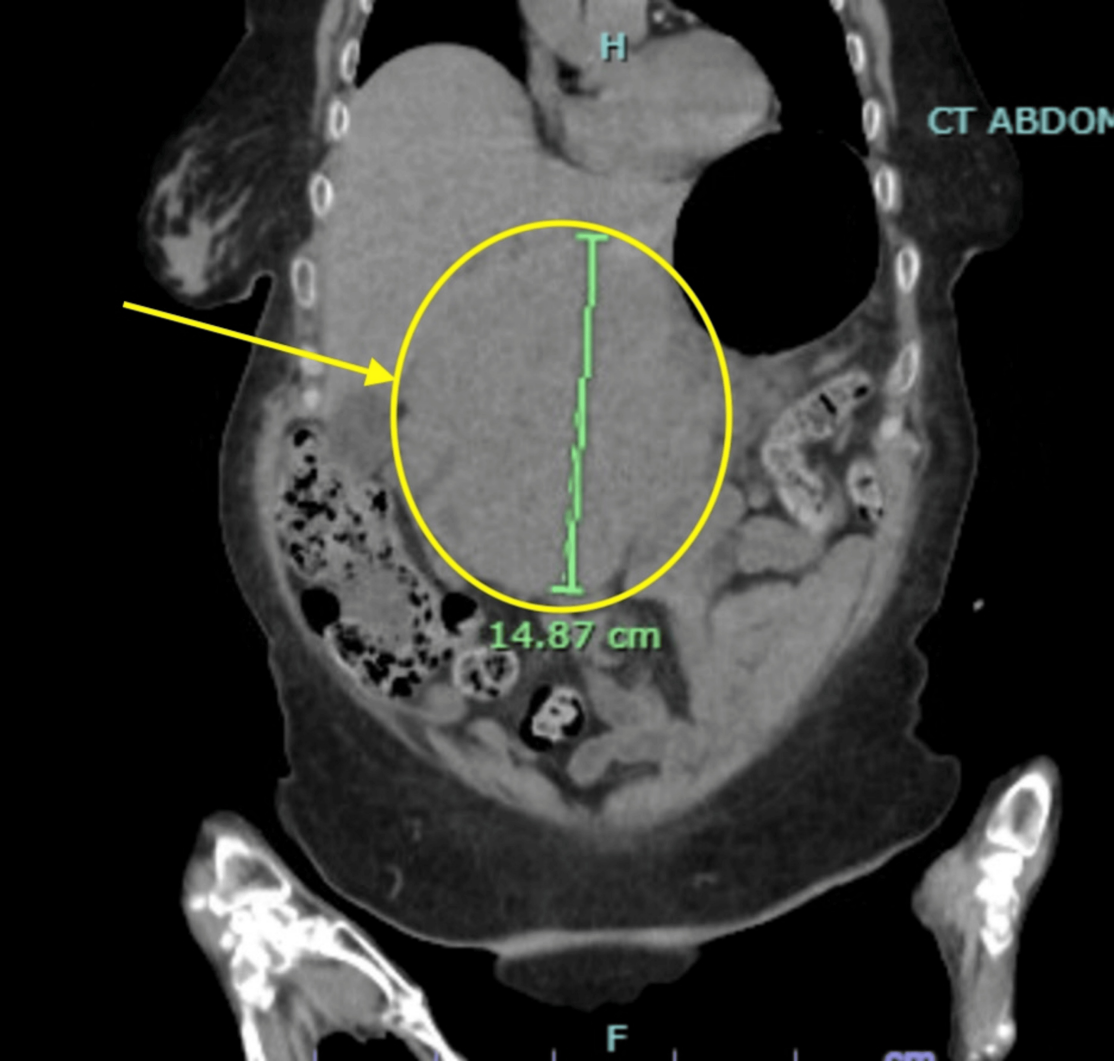
Coronal view of the initial CT of the chest, abdomen, and pelvis. The image displays a coronal cross-section from the patient’s CT scan of the chest, abdomen, and pelvis, obtained in the emergency department due to elevated liver function tests (see Table 2). The yellow circle and arrow indicate a large abdominal mass measuring a length of 14.87 cm. This is a different view of the same CT scan and the same mass indicated in Figure 1.

Due to her altered mental status, the patient underwent a non-contrast head CT scan, which was unremarkable. Elevated liver function tests were investigated further with a non-contrast CT of the abdomen and pelvis (CTAP), which revealed a 15.2 × 11.4 × 14.9 cm abdominal mass involving the head and body of the pancreas, notably without significant dilation of the pancreatic or biliary ducts. The mass also impinged on the duodenum but caused no gastric outlet obstruction. Evidence of suspicious metastatic disease was also present on initial CTAP, including a focal sclerotic lesion at the 12th thoracic vertebra (T12) as well as bilateral (R>L) adrenal masses (Figures 1, 2).

In the emergency department, the patient received a 1 L bolus of normal saline, one dose of piperacillin-tazobactam 3.375 g in D5W 50 mL, one dose of vancomycin 1 g in normal saline 250 mL, and potassium chloride 20 mEq in 100 mL.

Hospital course

**Figure 3:**
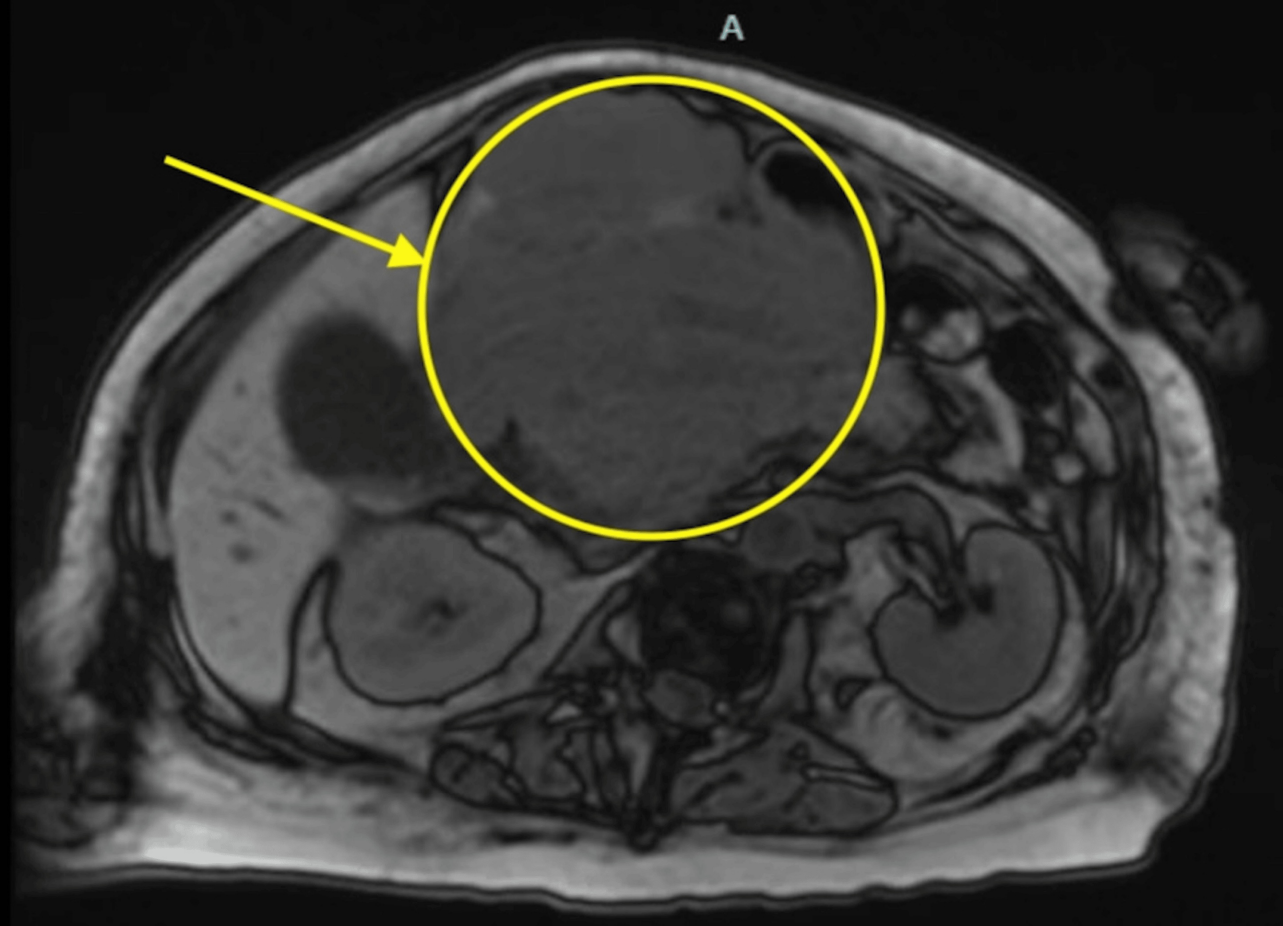
Axial view of magnetic resonance cholangiopancreatography. The image displays an axial cross-section of the patient’s magnetic resonance cholangiopancreatography scan taken during hospital admission to better visualize the origin of the abdominal mass. The yellow arrow and circle highlight the large abdominal mass, as seen on initial CT scan in Figures 1, 2.

**Figure 4:**
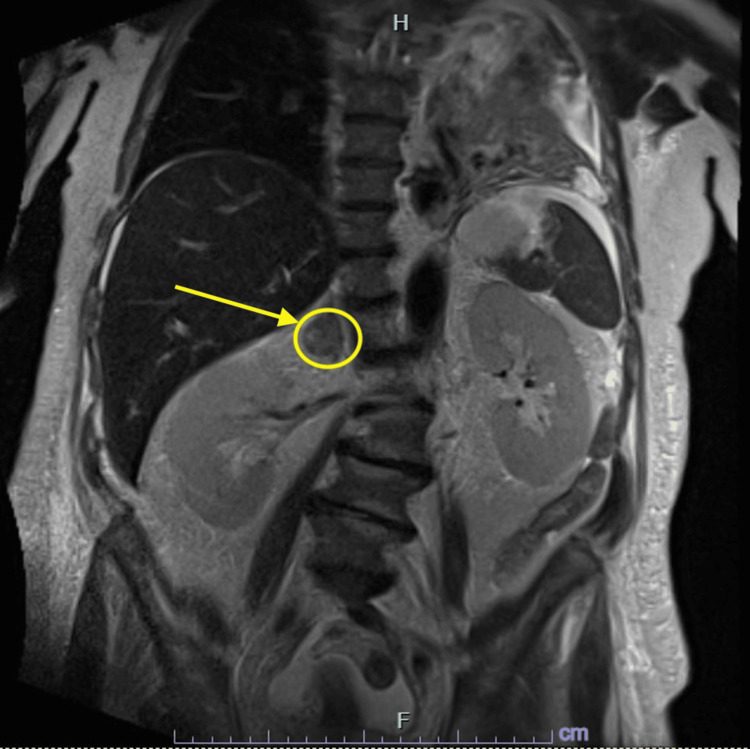
Coronal view of magnetic resonance cholangiopancreatography. The image displays a coronal cross-section of the patient’s magnetic resonance cholangiopancreatography scan taken during hospital admission. The yellow arrow and circle draw attention to a right adrenal nodule, which may be an incidental finding or suggestive of metastatic disease.

Follow-up MRI in the form of magnetic resonance cholangiopancreatography showed the large abdominal mass, a right adrenal mass, and a distended gallbladder, with no gallstones, gallbladder wall thickening, or common bile duct obstruction (Figures 3, 4).

Further workup included tumor marker studies, endocrine studies, ammonia, vitamin B12, folate, and a serum protein electrophoresis. Results are displayed in Table 3.

**Table 3:** Relevant subsequent laboratory results. The table displays the results of relevant subsequent workup conducted during the patient’s hospital stay. These include tumor markers, of which carcinoembryonic antigen and carbohydrate antigen 19-9 were negative and cancer antigen 125 was positive. Additionally, lactate dehydrogenase was elevated indicating rapid cell turnover, common in actively replicating cancers. Chromogranin A, a marker for neuroendocrine tumors, was also positive. These results suggest that the reason for this patient’s hypercalcemia is parathyroid hormone-related peptide (PTHrP) secretion, likely from her malignant tumor. This is supported by the elevated PTHrP, suppressed parathyroid hormone, and only slightly decreased Vitamin D. The other results in the table demonstrate negative endocrine workup for other types of functional tumors, evidenced by the normal catecholamine, metanephrine, and renin-angiotensin-aldosterone hormone levels. Additionally, the patient was found to have subclinical hypothyroidism, marked by elevated thyroid-stimulating hormome with a normal level of free thyroxine. Additionally, the patient was found to have normal levels of ammonia, vitamin B12, and folate, which further supports severe hypercalcemia as the primary cause of her altered mental status. Finally, the patient’s serum protein electrophoresis was not concerning for multiple myeloma. The abnormalities are more likely due to abnormal renal function in this patient.

Laboratory studies	Patient results	Reference values
Carcinoembryonic antigen	<2	Non-smoker: <2.5 ng/mL; smoker: <5.0 ng/mL
Carbohydrate antigen 19-9	<3	<34 U/mL
Lactate dehydrogenase	630 U/L	120–246 U/L
Uric acid	12.4 mg/dL	3.1–7.8 mg/dL
Cancer antigen 125	155 U/mL	<35 U/mL
Chromogranin A	841 ng/mL, 1,028 ng/mL	<311 ng/mL
Intact parathyroid hormone	10.6 pg/mL	18.4–88.0 pg/mL
Parathyroid hormone-related peptide	327 pg/mL, 891 pg/mL	11–20 pg/mL
Vitamin D 25 Hydroxy	27.1 ng/mL	30.0–100.0 ng/mL
Thyroid-stimulating hormone	5.7 µIU/mL	0.550–4.780 µIU/mL
Free thyroxine	1.21 ng/dL	0.89–1.76 ng/dL
Epinephrine	17 pg/mL	Supine: <58 pg/mL; upright: <82 pg/mL
Norepinephrine	337 pg/mL	Supine: 149-564 pg/mL; upright: 199-937 pg/mL
Dopamine	11 pg/mL	Supine: <16 pg/mL; upright: <27 pg/mL
Total Catecholamines	365 pg/mL	Supine: <632 pg/mL; upright: <1046 pg/mL
Plasma Renin Activity	1.88 ng/mL.hour	0.25–5.82 ng/mL.hour
Free metanephrine (plasma)	<25 pg/mL	57 pg/mL
Free Normetanephrine (plasma)	51	148 pg/mL
Free total metanephrines (plasma)	51	205 pg/mL
Aldosterone	<1 ng/dL	Upright 8:00–10:00 am = 28 ng/dL; upright 4:00–6:00 pm = 21 ng/dL; supine 8:00–10:00 am = 3–16 ng/dL
Random urine sodium	51 mmol/L	
Random urine creatinine	41.08 mg/dL	20.00–320.00 mg/dL
Ammonia	19 µmol/L	11–32 µmol/L
Vitamin B12	912 pg/mL	211–911 pg/mL
Folate	18.75 ng/mL	>5.38 ng/mL
*Serum protein electrophoresis*		
Protein, total	5.5 g/dL	6.0–8.0 g/dL
Albumin	2.95 g/dL	3.2–5.0 g/dL
Alpha 1 globulins	0.68 g/dL	0.1–0.4 g/dL
Alpha 2 globulins	0.87 g/dL	0.6–1.0 g/dL
Beta globulins	0.53 g/dL	0.6–1.30 g/dL
Gamma globulins	0.47 g/dL	0.70–1.50 g/dL
AG ratio	1.16	>1.00
Kappa Qt free light chains	29.40 mg/L	3.30–19.40 mg/L
Lambda Qt free light chains	13.72 mg/L	5.71–26.30 mg/L
Kappa-lambda free light chains ratio	2.14	0.26–1.65

The patient’s hypercalcemia was treated with aggressive hydration, calcitonin 300 units every 12 hours for a total of four doses, zoledronic acid 4 mg intravenously once, and emergent hemodialysis for two days. Hemodialysis was delivered through a central venous catheter in the right internal jugular vein, placed by vascular surgery. Improvement in her comprehensive metabolic panel is shown in Table 4.

**Table 4:** Comprehensive metabolic panel results from admission to discharge. The table displays the patient’s comprehensive metabolic panel results from her initial laboratory studies in the emergency department through her entire hospital admission until discharge. These results demonstrate the effectiveness of zoledronic acid given on 4/9, calcitonin given for on 4/8/25 and 4/9/25, and emergent hemodialysis received on 4/8/25 and 4/9/25 in improving the patient’s calcium levels from 21.6 mg/dL on admission to 11.5 mg/dL by 4/10/15, eventually reaching normal values later during the admission. These results also demonstrate resolution of acute kidney injury from an initial creatinine of 2.22 mg/dL to 0.70 mg/dL and overall improvement in liver function tests by discharge.

Date/Time	Reference values	4/7/25 15:17	4/8/25 04:54	4/8/25 10:50	4/8/25 22:55	4/9/25 04:50	4/9/25 17:39	4/10/25 06:20	4/10/25 18:53	4/11/25 03:41	4/11/25 18:43	4/12/25 04:55	4/13/25 05:18	4/14/25 06:22	4/15/25 05:33	4/16/25 05:17
Glucose, mg/dL	70–99	75	80	66	68	61	94	116	173	268	125	128	117	96	76	100
Sodium, mmol/L	136–145	140	139	139	140	142	139	141	144	142	142	140	141	139	142	143
Potassium, mmol/L	3.5–5.1	3.4	3.9	4.5	3.7	3.9	3.1	3.2	3.6	3.5	3.1	3.7	3.5	3.6	3.0	3.2
Chloride, mmol/L	98–107	102	105	107	104	106	103	103	107	106	106	105	105	104	103	108
CO_2_, mmol/L	20–31	27	25	17	26	25	28	25	24	23	25	23	26	21	25	25
Blood urea nitrogen, mg/dL	9–23	57	60	47	28	30	14	18	24	26	28	30	36	34	29	22
Creatinine, mg/dL	0.55–1.02	2.22	2.14	2.19	1.33	1.54	0.91	1.21	1.42	1.47	1.39	1.36	1.18	0.98	0.89	0.70
Estimated glomerular filtration rate, mL/min/1.73m^2^	>/= 60	24	25	24	44	37	>/= 60	49	41	39	42	43	51	>/= 60	>/= 60	>/= 60
Anion gap, mmol/L	5–15	11	9	15	10	11	8	13	13	13	11	12	10	14	14	10
Phosphorus, mg/dL	2.4–5.1		4.3			1.7		2.9						1.5		
Magnesium, mg/dL	1.6–2.6	2.71	2.62			1.87		1.58		1.51		1.55	1.43	1.31	1.40	1.28
Calcium, mg/dL	8.7-10.4	21.6	19.7, 19.6	18.9	13.5	13.6	10.5	11.5	11.7	10.9	10.8	10.4	9.8	9.4	8.2	8.1
Corrected calcium		22.4	20.6	19.8	14.6	14.6	11.8	12.8	13.1	12.3	12.1	11.6	11.2	10.6	9.4	9.4
Albumin, g/dL	3.4–5.0	3.0	2.7	2.9	2.6	2.8	2.4	2.4	2.3	2.3	2.4	2.5	2.2	2.5	2.5	2.4
AG ratio	>1.0	0.9	0.9	0.8	0.8	0.8	0.8	0.7	0.7	0.7	0.7	0.7	0.6	0.7	0.6, 0.6	0.7
Protein total, g/dL	6.0–8.0	6.5	5.8	6.4	6.0	6.2	5.5	5.7	5.5	5.7	6.0	5.9	5.6	6.1	6.6	5.9
Bilirubin total, mg/dL	0.2–1.3	0.6	0.9	0.7	0.7	0.7	1.5	1.5	0.8	0.5	0.6	0.5	0.4	0.3	0.4	0.4
Alkaline phosphatase, U/L	46–116	145	137	142	326	388	587	567	615	507	728	621	443	392	428	349
Aspartate aminotransferase, U/L	0–34	388	498	498	514	502	557	484	361	222	177	116	59	51	38	30
Alanine Aminotransferase, U/L	10–49	226	289	306	368	408	509	510	507	408	382	313	218	159	149	101

The patient’s mental status was formally assessed with regular mental status and neurologic examinations, which documented marked improvement in functioning following these treatments. Initial mental status and neurologic examinations demonstrated confusion, limited speech, inability to identify objects, limited answers to questions, and poor command following. In contrast, her examination on the day of discharge demonstrated that the patient was alert and oriented to person, place, and time and conversing appropriately with fluent speech.

During her hospitalization, the patient also underwent interventional radiology percutaneous biopsy of the abdominal mass, from which pathology revealed a well-differentiated NET. The tumor cells were arranged in highly vascularized trabeculae or nests and stained positive for AE1/3, synaptophysin, chromogranin (weak), and CD56. They stained negative for CK7, CK20, CDX2, GATA3, and TTF-1. The Ki-67 proliferative index was ~15%.

Pertinent outpatient follow-up

After discharge, the patient underwent an outpatient positron emission tomography scan for initial NET staging, which showed pathologic radiotracer uptake in the known abdominal mass, which was indistinguishable from the head of the pancreas. Additionally, there was uptake in a nodule located between the tail of the pancreas and the splenic hilum, thought to be a splenule, though an active lymph node could not be ruled out. Physiologic uptake of the radiotracer in the adrenal glands limited the evaluation of the right adrenal nodule, with further imaging recommended. Additionally, there was a new subpleural nodule in the right lower lung measuring 17 × 15 mm, which did not exhibit abnormal radiotracer uptake, though continued follow-up was recommended.

The patient continued to follow up as an outpatient with the oncology team and was started on lanreotide (somatuline) 120 mg every four weeks. The patient also followed up with the endocrinology team for monitoring of her adrenal nodule and nephrology for monitoring of her kidney function and fluid-electrolyte balance.

## Discussion

pNETs are a rare and heterogeneous group of neoplasms with diverse clinical presentations depending on functional status, tumor size, and metastatic burden [9]. While functional pNETs may produce distinct hormone-related syndromes, nonfunctional pNETs frequently present late, often as large masses with metastatic disease [5]. This case highlights an uncommon manifestation of a well-differentiated pNET with paraneoplastic hypercalcemia and altered mental status, an unusual and clinically significant presentation.

Hypercalcemia of malignancy is a well-established paraneoplastic syndrome and is most commonly mediated by the secretion of PTHrP. However, the secretion of PTHrP is typically associated with specific types of cancer, including squamous cell carcinoma, renal cell carcinoma, and breast cancer [13]. NETs rarely produce PTHrP. However, our patient came to medical attention due to acute metabolic encephalopathy and was subsequently found to have a markedly elevated serum calcium level (22.4 mg/dL) and PTHrP (327 pg/mL). Elevated liver enzymes prompted imaging evaluation with an abdominal CT, revealing a large mass arising from the pancreatic head. Therefore, paraneoplastic syndrome due to an underlying malignancy was suspected. Acute management was required due to the patient’s significantly altered mental status, which included intravenous hydration, bisphosphonates, calcitonin, and urgent hemodialysis. After several days of treatment with these aggressive measures, her calcium was corrected to a normal serum level, and with that came improvement in her mental status, which ultimately returned to baseline, underscoring the neurologic impact of this metabolic derangement [14].

Several case reports have been published in recent years regarding instances of refractory hypercalcemia in patients with pNETs. Many report the development of hypercalcemia several months or years after the initial diagnosis [4,7,15]. Others describe cases of pNETs with hypercalcemia present at the time of diagnosis [2,9,10,13,16-18]. There is also a wide range of symptom severity, severity of the calcium elevation, and treatments required to normalize calcium levels among these patients. This case adds to the literature, representing that great diversity in the time course, clinical symptoms, severity, and necessary interventions exists among patients with hypercalcemia of malignancy due to PTHrP-secreting pNETs.

## Conclusions

In summary, this case illustrates altered mental status due to paraneoplastic hypercalcemia as the initial manifestation of a pNET, emphasizing the importance of pNETs as part of the differential diagnosis of hypercalcemia. Early recognition and appropriate biochemical and histological workup are essential for prompt treatment initiation, which improves patient outcomes.
